# Effects of supplementation of sodium acetate on rumen fermentation and microbiota in postpartum dairy cows

**DOI:** 10.3389/fmicb.2022.1053503

**Published:** 2022-11-21

**Authors:** Zhiqiang Cheng, Zitong Meng, Dejin Tan, Osmond Datsomor, Kang Zhan, Miao Lin, Guoqi Zhao

**Affiliations:** ^1^Institute of Animal Culture Collection and Application, College of Animal Science and Technology, Yangzhou University, Yangzhou, China; ^2^Institutes of Agricultural Science and Technology Development, Yangzhou University, Yangzhou, China; ^3^Joint International Research Laboratory of Agriculture and Agri-Product Safety, The Ministry of Education of China, Yangzhou University, Yangzhou, China

**Keywords:** sodium acetate, bacterial community composition, rumen fermentation, negative energy balance, *Prevotella*

## Abstract

The primary product of rumen fermentation is acetic acid, and its sodium salt is an excellent energy source for post-partum cows to manage negative energy balance (NEB). However, it is unknown how adding sodium acetate (NAc) may affect the rumen bacterial population of post-partum cows. Using the identical nutritional total mixed ration (TMR), this research sought to characterize the impact of NAc supplementation on rumen fermentation and the composition of bacterial communities in post-partum cows. After calving, 24 cows were randomly assigned to two groups of 12 cows each: a control group (CON) and a NAc group (ACE). All cows were fed the same basal TMR with 468 g/d NaCl added to the TMR for the CON group and 656 g/d NAc added to the TMR for the ACE group for 21 days after calving. Ruminal fluid was collected before morning feeding on the last day of the feeding period and analyzed for rumen bacterial community composition by 16S rRNA gene sequencing. Under the identical TMR diet conditions, NAc supplementation did not change rumen pH but increased ammonia nitrogen (NH_3_-N) levels and microbial crude protein (MCP) concentrations. The administration of NAc to the feed upregulated rumen concentrations of total volatile fatty acids (TVFA), acetic, propionic, isovaleric and isobutyric acids without affecting the molar ratio of VFAs. In the two experimental groups, the *Bacteroidota*, *Firmicutes*, *Patescibacteria* and *Proteobacteria* were the dominant rumen phylum, and *Prevotella* was the dominant rumen genus. The administration of NAc had no significant influence on the α-diversity of the rumen bacterial community but upregulated the relative abundance of *Prevotella* and downregulated the relative abundance of *RF39* and *Clostridia_UCG_014*. In conclusion, the NAc supplementation in the post-peripartum period altered rumen flora structure and thus improved rumen fermentation in dairy cows. Our findings provide a reference for the addition of sodium acetate to alleviate NEB in cows during the late perinatal period.

## Introduction

After calving, dairy cows in the transition period are subjected to various physiological stress shocks that cause low dry matter intake, hormonal changes, and high morbidity (Contreras et al., 2018). Especially in high-performance dairy cows, decreased feed intake makes it impossible to fulfill the nutritional needs of rapidly producing milk, resulting in development of NEB (Lopreiato et al., 2020). Adipose tissue is catabolized into considerable amounts of non-esterified fatty acids (NEFA), which subsequently enter the liver. This will swiftly lead to the development of ketosis or fatty liver, which will reduce liver and immune function in post-partum cows (Gao et al., 2018; White, 2020). Additionally, the body’s metabolism is boosted, and a significant amount of reactive oxygen species and lipid peroxides are created in the NEB state to adjust to breastfeeding requirements. This puts cows in a condition of oxidative stress that surpasses the body’s antioxidant system’s capacity for scavenging, which causes immunosuppression and in turn causes metabolic illnesses such as mastitis, metritis, retained placenta, and reduced fertility (Ingvartsen and Moyes, 2013). Therefore, the intensity and length of NEB have a significant impact on cow health, making them more prone to illness and less effective in terms of milk output and quality, which in turn has a negative impact on farm economics.

As a frequent treatment for NEB, exogenous nutritional addition to modulating dairy cow metabolism during the perinatal period can help dairy cows produce more milk, eliminate energy metabolism problems, and enhance breeding effectiveness (Sordillo and Raphael, 2013; Lopreiato et al., 2020). Acetic acid, which typically makes up around 60% of fermented acid products in ruminants, is crucial for the synthesis of milk fat and energy supply as it serves as a primary precursor to milk fat and a basic energy source (Aschenbach et al., 2010). Sodium acetate (NAc) can be utilized to treat NEB issues in dairy cows by hydrolyzing into lactic acid and sodium ions in the rumen (Bampidis et al., 2021). According to the experimental results of injecting 0, 5, 10, and 15 mol/d of acetic acid into the rumen, dairy cows’ ability to produce milk and milk fat increased linearly (Urrutia and Harvatine, 2017). In addition, reports have been made using intraruminal infusion of acetic acid or NAc to test its effects on milk production and milk composition and feed intake in lactating dairy cows (Rook and Balch, 1961; Urrutia and Harvatine, 2017). Although intraruminal infusion of acetic acid has been proven to positively impact energy balance, body health, milk output, and milk composition in nursing dairy cows. The effectiveness of acetic acid to treat NEB of post-partum cows as a result of inadequate feed intake has not been documented, although the putative role of acetic acid in the energy metabolism of cows is well recognized. We postulated that adding acetic acid to TMR after calving may help cows to produce more milk, boost immunity, and successfully treat NEB. By including an additional 8 mol/d of NAc in the feed of post-calving cows, we confirmed this theory (unpublished data). Whereas, uncertainty persists about the part rumen microbes play in the supplementary NAc-induced NEB reduction.

A wide range of microorganisms are engaged in the deconstruction and use of feed to supply the nutritional needs of ruminants for maintenance, development, and lactation through fermentation of feed in the rumen of ruminants, which is a sizable “fermenter” (Bickhart and Weimer, 2018). The rumen microbiome, which is sensitive to changes in feed type and diet structure, is substantially connected with dairy cow performance (Liu et al., 2019). The health and productivity of dairy cows can be controlled by modifying the makeup of the rumen’s microbial population and fermentation products. It is critical to understand the alterations in the makeup of the rumen microbial population of dairy cows in order to explore the influence of NAc feed additives on the rumen microorganisms of dairy cows. Because acetate is one of the principal fermentation products of the rumen microflora, it may modify rumen fermentation indicators and rumen bacterial community composition, lowering NEB in post-partum cows. Based on this, we hypothesize that NAc may improve post-partum cows performance by altering rumen fermentation and microbiota. As a result, the purpose of this study was to use 16S rRNA sequencing to investigate the impact of NAc supplementation on rumen fermentation and rumen bacterial community composition in dairy cows following calving.

## Materials and methods

All Holstein dairy bovines utilized in this think were entirely cared for in understanding the standards of Yangzhou University, the Institutional Animal Care and Use Committee (SYXK (Su) IACUC 2016-0019).

### Experimental design, animals, diets, and management

The exploration was conducted from September to December 2021 at the test base of The Animal Nutrition and Feed Engineering Research Center of Yangzhou University (Gaoyou, China; yoke stall). Twenty-four Holstein cows in postpartum were randomly divided into two blocks (*n* = 12 per treatment) based on parity (2, *n* = 24), weight (640.1 ± 20.9 kg), and anticipated calving date. All cows were nourished the equal basal total mixed ration diet (TMR), formulated based on the nutritional requirement of the National Research Council (NRC, 2001). The composition and nutrient level of the diets are given in Table 1. Similar to previous research (Urrutia et al., 2019), the control and NAc groups were given 468 g/day sodium chloride (Jiangsu Baimei Chemical Co., Ltd.) and 656 g/day sodium acetate (Shandong Yutian Food Science & Technology Co., Ltd.) with >99% effective constituent content from calving to 21 days postpartum, respectively. The control group (sodium chloride) was designed to provide equal moles of sodium as the sodium acetate group. The oral drenching was achieved by separately taking an equal portion from the offered TMR (Ensure 5% ~ 10% orts daily), mixing it with sodium chloride or sodium acetate using a mixer and then orally delivering (drenched) to each cow within the experimental groups (control or NAc group). Afterwards, the cows had access to the remaining portion of the TMR diet offered. Cows were drenched and fed TMR three times a day at 08:00, 14: 00 and 21: 00. The cows had free access to water throughout the experimental period. The daily offered TMR feed throughout the experimental period was adjusted based on the daily leftover from each cow. The feed samples were collected from the trough on three consecutive days per week, and one sample was mixed each week. The nutrient content was assayed in previous studies (Jiang et al., 2022).

**Table 1:** Composition and nutrient levels of the experiment diet [dry matter (DM) basis %].

Ingredients	Content
Alfalfa	8.39
Oat grass	6.43
Whole corn silage	52.18
Corn	8.1
Soya bean meal	10.8
Cotton seed meal	6.8
DDGS	4.8
Premix^1^	2.5
Total	100
*Nutrient levels*	
CP	16.47
EE	3.84
NDF	33.06
ADF	20.25
Ash	7.28
Calcium	0.74
Phosphorus	0.35
NEL(MJ/kg)^2^	3.25

DDGS, distiller dried grains with soluble; CP, crude protein; EE, ether extract; NDF, neutral detergent fiber; ADF, acid detergent fiber.

1 Per kilogram of premix provide 55 mg of Mn as MnSO_4_; 62.5 mg of Zn as ZnSO_4_; 0.55 mg of Se as Na_2_SeO_3_; 75 mg of Fe as Fe_2_ (SO_4_)_3_; 32 mg of Cu as CuSO_2_; 1.00 mg of I as KI; 0.40 mg of Co as CoCl_2_; 1,100,000 IU of Vitamin A; 270,000 IU of Vitamin D; 200,000 mg of Vitamin E; 4,200 mg of Vitamin K_3_.

2 Predicted values from NRC (2001) model.

### Ruminal sample collection and analyses

Ruminal contents samples were collected with an oral collector (A1141K, Colibri Pastoral Technology Co., Wuhan, Hubei, China) before morning feeding (At 07: 30) on day 21 of parturition, and ruminal fluid was collected from a total of 24 cows. Among them, the insertion length of the rumen fluid collector is about two meters. The first 150 ml of rumen contents were discarded to avoid saliva contamination. Then 150 ml of rumen contents were collected after filtering through 4 layers of sterile gauze and dispensed into ten 15 ml plastic cryotubes. Ruminal pH was immediately with a mettler-toledo pH meter (Model FE20, Shanghai, China). The NH_3_-N, VFA and MCP concentrations were determined using the methods of previous studies (Zhang et al., 2022). The rumen content samples were kept in liquid nitrogen to quickly cool down and then stored at −80°C for later microbiome analysis (Tables 2, 3).

**Table 2:** Effect of NAc addition on rumen pH, NH_3_-N and volatile fatty acids (VFA).

Items	Treatments^1^		*p*-value
	CON	ACE	
pH	6.90 ± 0.06	6.77 ± 0.05	0.110
NH_3_-N, mg/dL	10.21 ± 0.76	11.07 ± 0.0.71	0.418
MCP, mg/dL	44.33 ± 1.64	55.07 ± 0.92	<0.001
**Total VFA, mmol/L**	82.48 ± 3.34	103.47 ± 4.02	0.001
Acetate, mmol/L	57.61 ± 2.10	72.90 ± 2.79	<0.001
Propionate, mmol/L	13.98 ± 0.47	17.12 ± 0.71	0.001
Iso-butyrate, mmol/L	0.29 ± 0.01	0.35 ± 0.02	0.008
Butyrate, mmol/L	8.64 ± 0.87	10.67 ± 0.58	0.066
Iso-valerate, mmol/L	0.97 ± 0.10	1.26 ± 0.07	0.025
Valerate, mmol/L	0.98 ± 0.08	1.16 ± 0.05	0.059
Acetate/propionate	4.14 ± 0.13	4.28 ± 0.10	0.430

NH_3_-N = ammonia-N, MCP = microbial crude protein.

1 Treatments: CON = control group, basal diet plus 468 g/day oral sodium chloride; ACE = NAc group, basal diet plus 656 g/day oral NAc.

**Table 3:** Effects of dietary NAc supplementation on α-diversity indexes of rumen bacteria.

Items	Treatments^1^		*p*-value
	CON	ACE	
chao1	1233.93 ± 40.98	1174.37 ± 29.38	0.250
observed_otus	1229.25 ± 40.97	1171.08 ± 29.75	0.263
shannon	8.94 ± 0.24	7.91 ± 0.12	0.487
simpson	0.973 ± 0.013	0.981 ± 0.003	0.594
pielou_e	0.789 ± 0.021	0.776 ± 0.010	0.581

1 Treatments: CON = control group, basal diet plus 468 g/day oral sodium chloride; ACE = NAc group, basal diet plus 656 g/day oral NAc.

### 16S rRNA gene sequencing and data processing

The total DNA of rumen bacteria were extracted by the cetyltrimethylammonium bromide (CTAB) and then assessed for DNA quality by 1% agarose gel electrophoresis. The V4 high variant region of the bacteria was amplified using primers 515F (5′-GTGCCAGCMGCCGCGGTAA-3′) and 806R (5′-GGACTACNNGGGTATCTAAT-3′; Lin et al., 2021). The PCR products were sent to Novogene Co., Ltd. (Beijing) for high-throughput sequencing analysis.

Data read from both ends of the raw DNA fragments were processed using FASTP (Version 0.14.1; Chen et al., 2018). The final amplicon sequence variants (ASVs), as well as feature tables, were obtained by noise reduction using the DADA2 module in QIIME2 (Version 1.9.1) software or deblur based on the quality-controlled Effective Tags and filtering out sequences with abundance less than 5 (Bolyen et al., 2019). Subsequently, the ASVs were compared with the SILVA (version 138.1) database to determine species information (Quast et al., 2012). Based on the ASVs information, α-diversity analysis (chao1, observed_otus, shannon, simpson, pielou_e) was calculated using QIIME2. Principal coordinate analysis (PCoA), non-metric multidimensional scale analysis (NMSD) and LDA Effect Size (Lefse) were analyzed using online resources.^1^ The 16S sequence data have been submitted to the GSA database under the PRJCA010405 program.

### Statistical analysis

The correlation and differences between rumen fermentation parameters and bacterial abundance were analyzed with Spearman’s correlation analysis and independent samples t-tests in SPSS 26 (IBM, New York, USA) and results were expressed as mean ± SEM. Correlation coefficients with absolute values greater than 0.5 are considered relevant. Significant differences were declared at *p* < 0.05. And *p* < 0.01 was considered a highly significant difference.

## Results

### Ruminal fermentation parameters

The NAc supplementation significantly increased rumen microbial crude protein (MCP) concentration (*p* < 0.001), but had no significance on pH (*p* = 0.110) and NH_3_-N concentration (*p* = 0.418). Dietary addition of NAc significantly increased the concentrations of acetate (*p* < 0.001), propionate (*p* = 0.001), isobutyrate (*p* = 0.008), isovalerate (*p* = 0.025) and TVFA (*p* = 0.001) in the rumen fluid, but did not affect the ratio of acetate to propionate (Table 2). NAc supplementation induced an increasing trend in the concentration of both butyrate (*p* = 0.066) and valerate (*p* = 0.059). The molar ratios of individual rumen fluid VFA are shown in Supplementary Table S1. The addition of NAc did not alter the molar percentage of rumen fluid VFA.

### Sequencing and diversity measures

**Figure 1:**
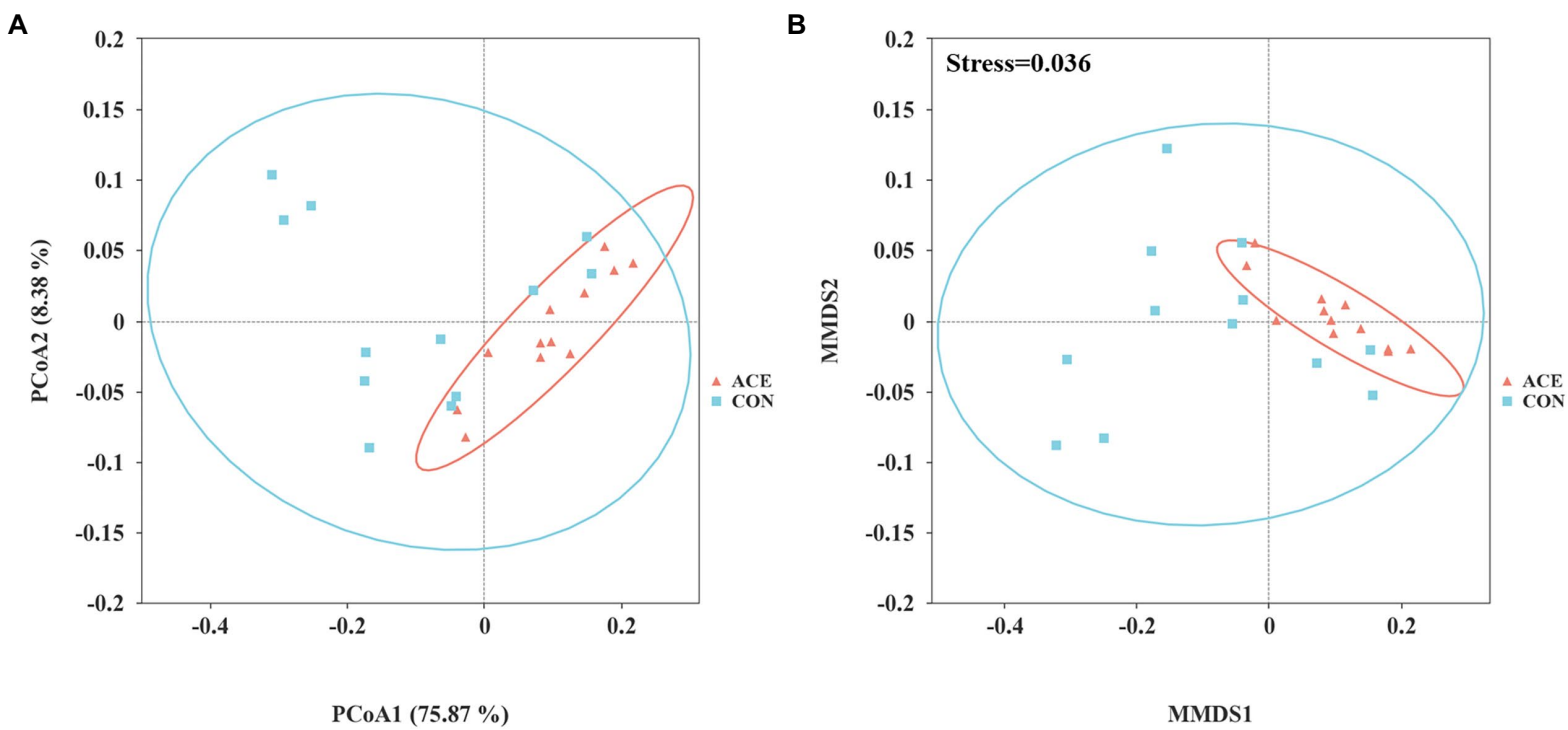
Beta community diversity analysis of the CON and ACE groups of rumen microbiota. **(A)** Principal coordinate analysis (PCoA) and **(B)** non-metric multidimensional scale analysis (NMDS) CON = control group, basal diet plus 468 g/day oral sodium chloride; ACE = NAc group, basal diet plus 656 g/day oral NAc.

A total of 1,927,895 high-quality V4 16S rRNA sequences were generated in 24 rumen samples, with an average of 80,329 per sample (minimum 71,936; maximum 88,726). A total of 5,499 ASVs were detected in the samples, with 4,364 and 3,613 detected in the CON and ACE groups, respectively. Venn diagrams (Supplementary Figure S1) show the number of common and unique rumen bacterial ASVs in the two groups. The sparsity curves of the rumen samples (Supplementary Figure S2) indicate that the sequencing depth was sufficient to assess the different bacterial communities accurately. The supplementation of NAc did not affect (*p* > 0.05) the α-diversity (Table 3) measurement index (chao1, observed_otus, shannon, simpson, pielou_e). As shown in the PCoA and NMDS plots, the distribution of samples from the ACE group was relatively concentrated, and the two groups crossed and overlapped each other (Figure 1). This indicates that the samples from the ACE group had better reproducibility and did not differ significantly from the rumen microbial community of the CON group in terms of β-diversity.

### Microbiota composition

**Figure 2:**
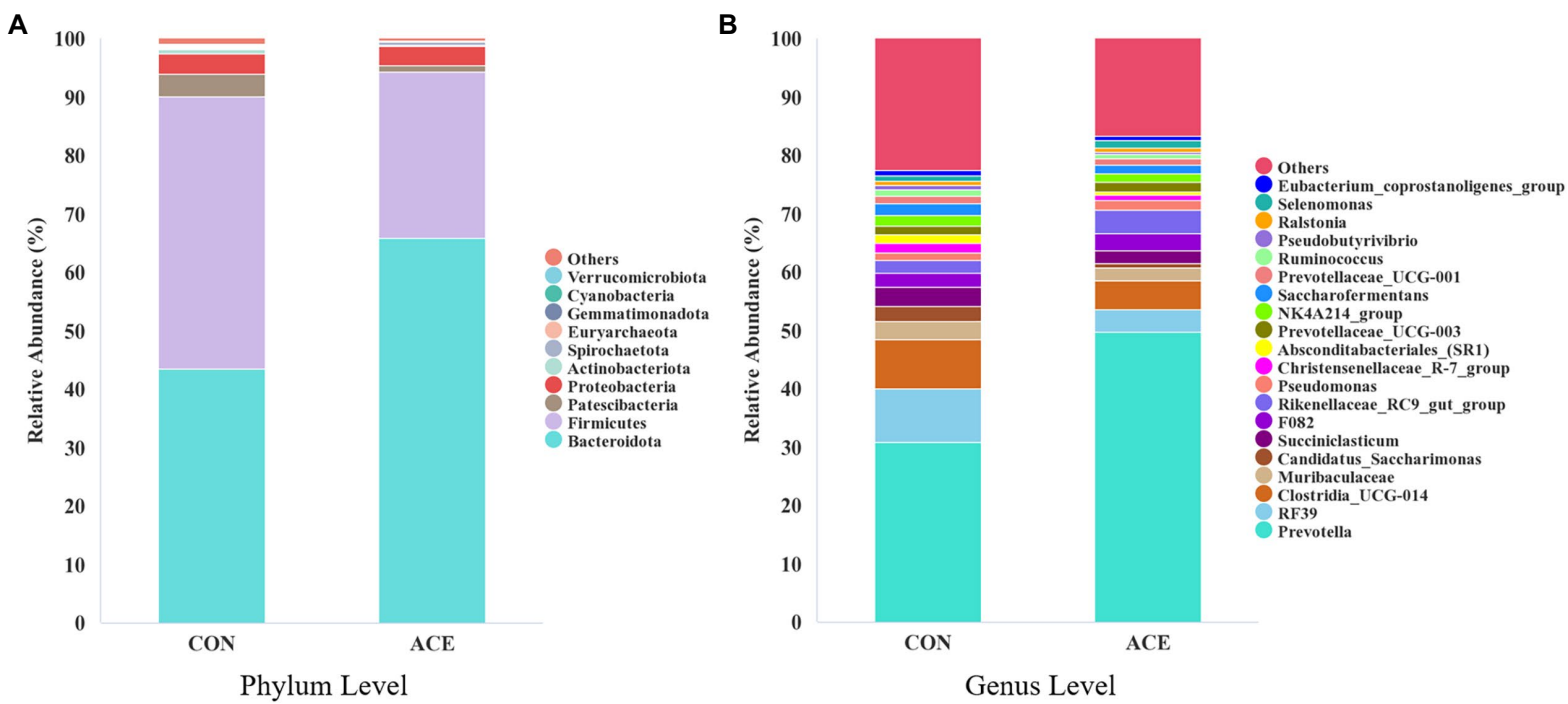
Distribution of rumen bacterial community composition in CON and ACE groups. **(A)** Phylum level; **(B)** genus level. CON = control group, basal diet plus 468 g/day oral sodium chloride; ACE = NAc group, basal diet plus 656 g/day oral NAc.

The effect of NAc addition on bacterial composition of the rumen was compared by taxonomic analysis. A total of 28 bacterial phyla were obtained from 24 rumen samples. Figure 2A shows that the *Bacteroidota* (CON: 43.54%; ACE: 65.82%), *Firmicutes* (CON: 46.47%; ACE: 28.43%), *Patescibacteria* (CON: 4.02%; ACE: 1.25%) and *Proteobacteria* (CON: 3.41%; ACE: 3.29%) were the major clades in all groups. The addition of NAc increased the relative abundance of *Bacteroidota* in the rumen (*p* = 0.001) but decreased the abundance of *Firmicutes* (*p* = 0.001) and *Patescibacteria* (*p* < 0.01; Table 4). At the genus level, a total of 388 genera were detected in the rumen. As shown in Figure 2B and Table 5, the unclassified sequences in the CON and ACE groups were 9.82 and 7.48%, respectively, with *Prevotella* (CON: 30.73%; ACE: 49.71%) being the most abundant genus in the rumen. The results of this experiment showed that the addition of NAc to the diet increased the abundance of *Prevotella* (*p* < 0.01) and *Rikenellaceae_RC9_gut_group* (*p* = 0.001) in *Bacteroidota*. However, NAc supplementation reduced the abundance of *RF39* (*p* < 0.05), *Clostridia_UCG-014* (*p* < 0.05), *Saccharofermentans* (*p* < 0.05), *Pseudobutyrivibrio* (*p* < 0.05), *Lachnospira* (*p* < 0.05), *Eubacterium_ruminantium_group* (*p* < 0.05), *Butyrivibrio* (*p* < 0.05) and *Anaerovibrio* (*p* < 0.05) in *Firmicutes.* Besides, the abundance of *Candidatus_Saccharimonas* (*p* < 0.05) and *Absconditabacteriales_(SR1)* (*p* < 0.05) in the *Patescibacteria* of the ACE group of rumen microorganisms were reduced.

**Table 4:** Effects of dietary supplementation with NAc on the relative abundance of ruminal bacterial communities at phylum level (average relative abundance ≥0.5% for at least one treatment) of dairy cows.

Phylum	Treatments^1^		*P*-value
	CON	ACE	
*Bacteroidota*	43.54 ± 5.09	65.82 ± 9.47	0.001
*Firmicutes*	46.47 ± 3.95	28.43 ± 7.54	0.001
*Patescibacteria*	4.02 ± 0.85	1.25 ± 0.15	0.008
*Proteobacteria*	3.41 ± 0.71	3.29 ± 0.86	0.918
*Actinobacteriota*	0.68 ± 0.16	0.20 ± 0.03	0.014

1 Treatments: CON = control group, basal diet plus 468 g/day oral sodium chloride; ACE = NAc group, basal diet plus 656 g/day oral NAc.

**Table 5:** Effects of dietary supplementation with NAc on the relative abundance of ruminal bacterial communities at genus level (average relative abundance ≥0.5% for at least one treatment) of dairy cows.

Phylum	Genus	Treatments^1^		*P*-value
		CON	ACE	
*Bacteroidota*	*Prevotella*	30.73 ± 5.10	49.71 ± 3.21	0.005
	*Muribaculaceae*	3.09 ± 0.81	2.18 ± 0.32	0.311
	*F082*	2.23 ± 0.50	2.99 ± 0.24	0.18
	*Rikenellaceae_RC9_gut_group*	2.27 ± 0.24	3.94 ± 0.34	0.001
	*Prevotellaceae_UCG-003*	1.41 ± 0.21	1.69 ± 0.16	0.287
	*Prevotellaceae_UCG-001*	1.24 ± 0.22	1.22 ± 0.14	0.927
*Firmicutes*	*RF39*	9.25 ± 1.78	3.94 ± 0.31	0.013
	*Clostridia_UCG-014*	8.48 ± 1.40	4.92 ± 0.61	0.035
	*Succiniclasticum*	3.46 ± 0.58	2.19 ± 0.40	0.087
	*Christensenellaceae_R-7_group*	1.66 ± 0.32	1.01 ± 0.12	0.077
	*NK4A214_group*	1.92 ± 0.26	1.39 ± 0.23	0.135
	*Saccharofermentans*	2.03 ± 0.16	1.40 ± 0.17	0.015
	*Ruminococcus*	1.09 ± 0.19	0.75 ± 0.10	0.122
	*Pseudobutyrivibrio*	0.82 ± 0.19	0.35 ± 0.04	0.034
	*Selenomonas*	0.93 ± 0.18	1.32 ± 0.16	0.123
	*Eubacterium_coprostanoligenes_group*	0.95 ± 0.14	0.63 ± 0.08	0.054
	*Lachnospira*	0.64 ± 0.12	0.34 ± 0.03	0.03
	*Eubacterium_ruminantium_group*	0.80 ± 0.09	0.56 ± 0.07	0.048
	*Butyrivibrio*	0.61 ± 0.11	0.28 ± 0.04	0.012
	*Anaerovibrio*	0.49 ± 0.07	0.57 ± 0.07	0.468
*Patescibacteria*	*Candidatus_Saccharimonas*	2.52 ± 0.67	0.73 ± 0.15	0.023
	*Absconditabacteriales_(SR1)*	1.50 ± 0.37	0.51 ± 0.12	0.026
*Proteobacteria*	*Pseudomonas*	1.23 ± 0.37	1.66 ± 0.50	0.489
	*Ralstonia*	0.69 ± 0.22	0.69 ± 0.24	0.991

1 Treatments: CON = control group, basal diet plus 468 g/day oral sodium chloride; ACE = NAc group, basal diet plus 656 g/day oral NAc.

**Figure 3:**
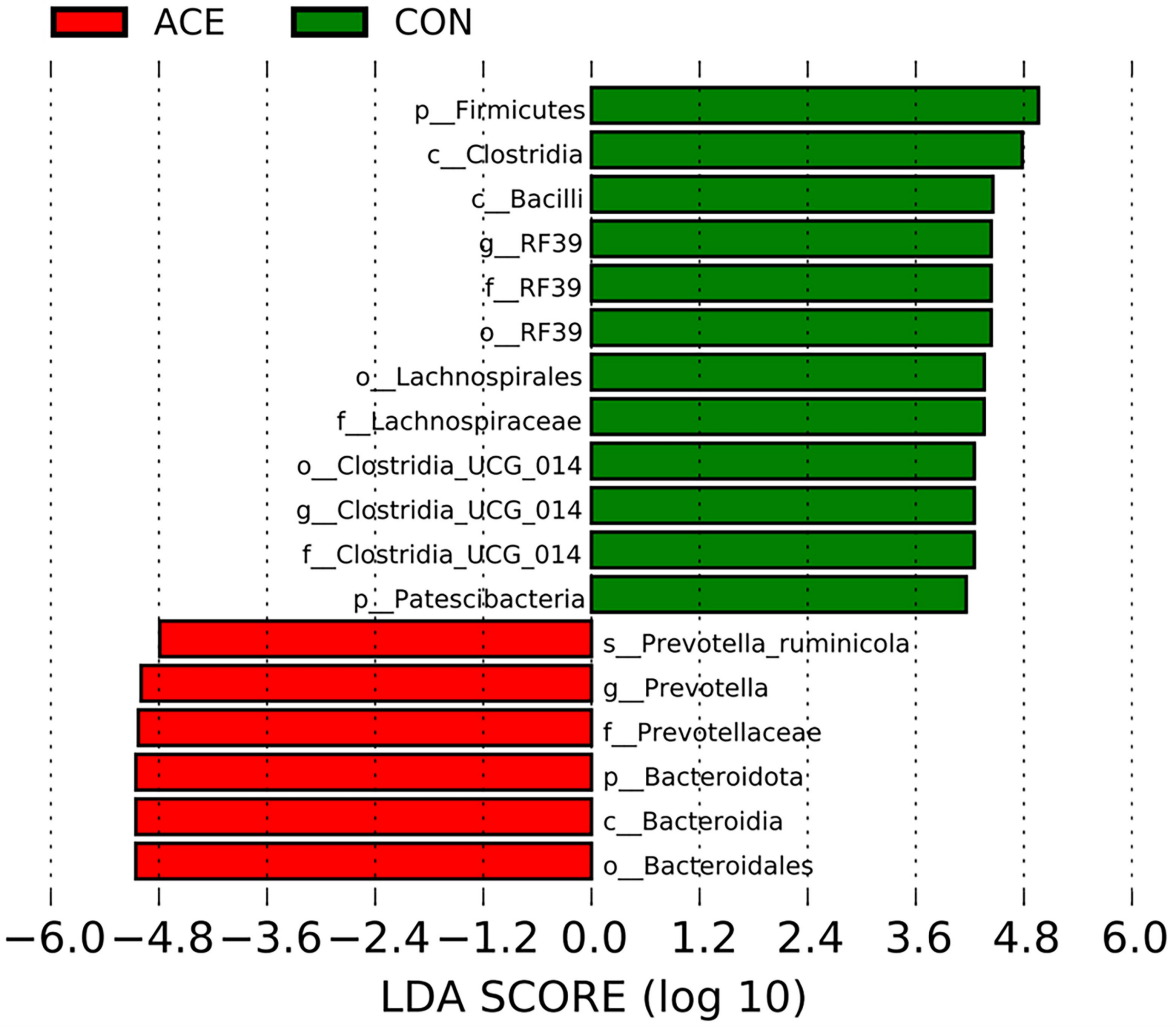
Histograms of LDA scores >4 were calculated for each taxonomic unit from phylum to genus. LDA scores indicate differences in relative abundance between two communities, with an exponential multiplicative change of 10 indicating significant differences between taxa.

**Figure 4:**
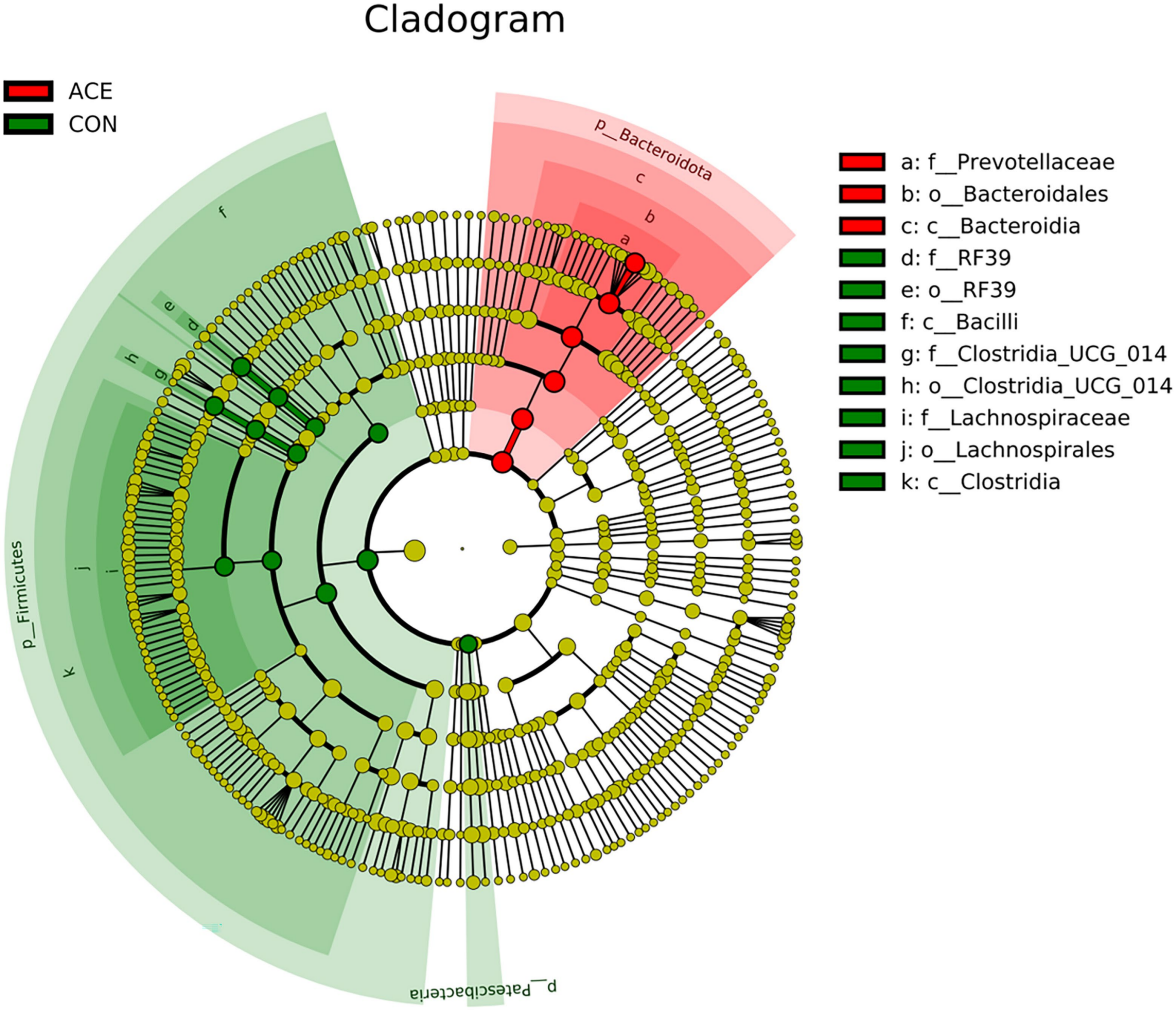
Linear discriminant analysis effect size (LEfSe) analysis to identify biomarker genera within the rumen microbial community of diets supplemented with NAc. Nodes indicate the position of microbial groups within taxa, and circles and axes indicate the number of taxa at the level of the taxon and at lower levels. Letters provided prior to the microbial taxa associated with the taxonomic level (p_: phylum; c_: class; o_: order; f_: family; g_: genus).

LEfSe analysis was performed using all microbial data to identify key bacterial groups associated with the diets and NAc addition. Figure 3 depicts a representative structural branched map of the major microbial groups, showing the most significant differences in taxa between treatments. Figure 4 shows the differences in the abundance of the different bacterial groups in CON and ACE. Among them, the bacterial genera identified as biomarkers in the CON group were *RF39* and *Clostridia_UCG-014*, and the bacterial genus with the greatest difference in the ACE group was *Prevotella*.

### Correlation analysis of rumen fermentation parameters and major bacteria

**Figure 5:**
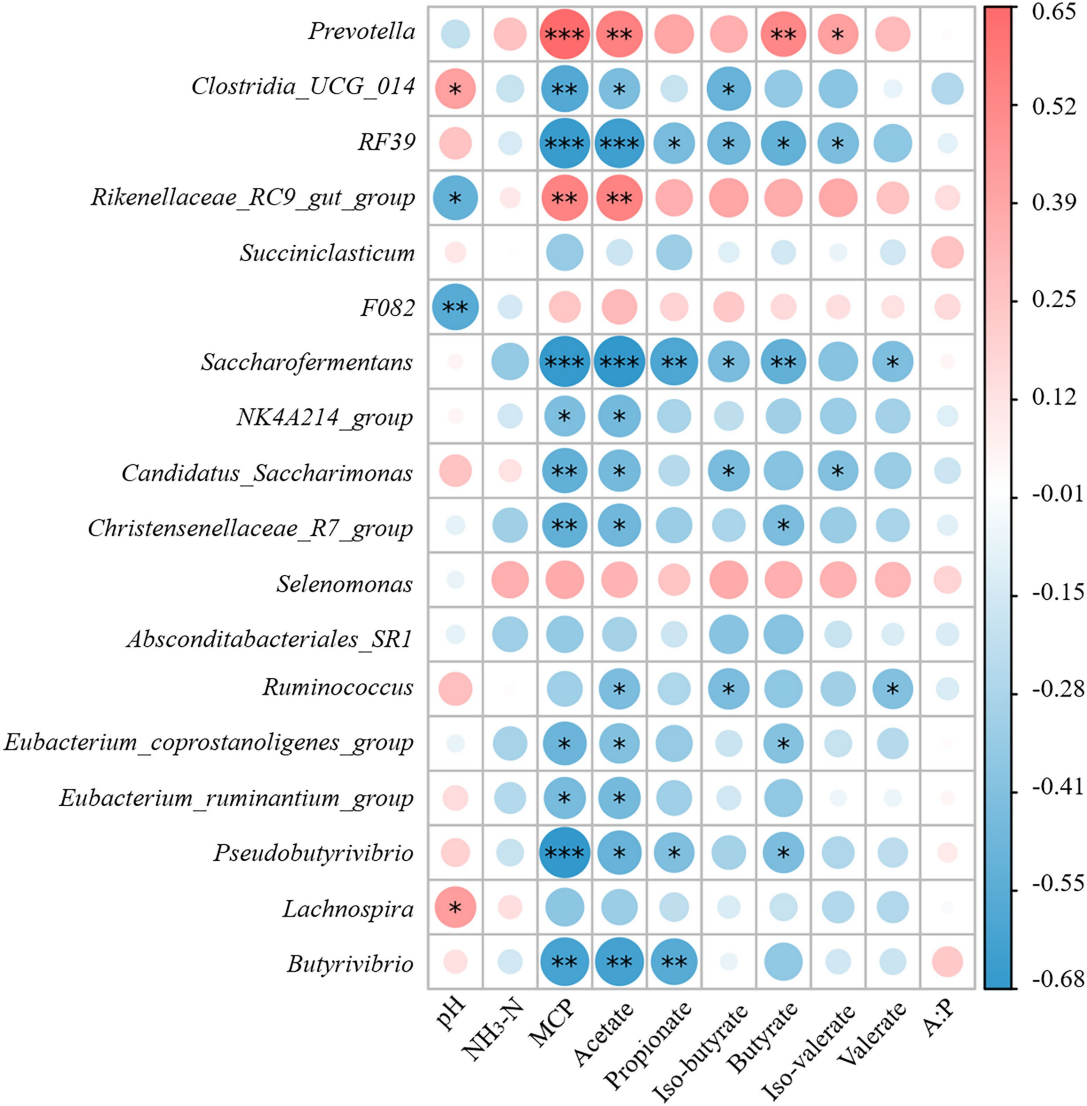
Spearman correlation between rumen fermentation indicators and bacterial populations. The area of the circle indicates the magnitude of the correlation, and the different colors indicate positive correlation (red) or negative correlation (blue). * indicates 0.01 < *p* ≤ 0.05, ** indicates *p* ≤ 0.01 and *** indicates *p* ≤ 0.001.

Spearman correlation analysis was performed to assess the relationship between rumen fermentation parameters and rumen microflora structure in the presence of NAc addition. At the genus level, pH was negatively correlated with the relative abundance of *F082* (*r* = −0.56; *p* < 0.01) and *Rikenellaceae_RC9_gut_group* (*r* = −0.51; *p* < 0.05). The relative abundance of *Prevotella* in rumen fluid was positively correlated with the concentrations of MCP (*r* = 0.65; *p* < 0.001), Acetate (*r* = 0.56; *p* = 0.005), and Butyrate (*r* = 0.53; *p* = 0.008). The relative abundance of *Rikenellaceae_RC9_gut_group* had a positive correlation with the concentrations of MCP (*r* = 0.55; *p* < 0.01) and Acetate (*r* = 0.55; *p* < 0.01) similar to that of *Prevotella*. In contrast, the relative abundance of *RF39* was negatively correlated with the concentrations of MCP (*r* = −0.66; *p* < 0.001), Acetate (*r* = −0.64; *p* < 0.001), and Butyrate (*r* = −0.51; *p* < 0.05). Besides, the relative abundance of *Saccharofermentans* was negatively correlated with the concentrations of MCP (*r* = −0.67; *p* < 0.001), Acetate (*r* = −0.68; *p* < 0.001), Propionate (*r* = −0.59; *p* < 0.01) and Butyrate (*r* = −0.52; *p* < 0.01) concentrations were also negatively correlated. But the concentration of MCP was positively correlated with the concentrations of *Clostridia_UCG_014* (*r* = −0.57; *p* < 0.01), *Candidatus_Saccharimonas* (*r* = −0.52; *p* < 0.01), *Christensenellaceae_ R7_group* (*r* = −0.52; *p* < 0.01) and *Pseudobutyrivibrio* (*r* = −0.67; *p* < 0.001) were negatively correlated in relative abundance. Other negative correlations included between *Butyrivibrio* and MCP (*r* = −0.61; *p* < 0.01), acetate (*r* = −0.61; *p* < 0.01), and propionate (*r* = −0.56; *p* < 0.01; Figure 5.)

## Discussion

Acetate is the main VFA produced in the rumen, providing 50% of the energy produced by VFA metabolism and is the main source of energy and substrate for milk fat synthesis in dairy cows (Baldwin and Smith, 1971; Bergman, 1990; Urrutia et al., 2019). Numerous studies have shown that rumen infusion of acetic acid or NAc has a positive effect on milk production, milk composition and especially milk fat synthesis in lactating cows (Rook and Balch, 1961; Reynolds et al., 1979; Urrutia and Harvatine, 2017; Matamoros et al., 2021). However, there is little research on the combined effects of NAc as a supplement to diets on cows in the post-peripartum period. This study was part of a larger experiment to investigate the effects of NAc supplementation in the diet on feed intake, body weight changes, rumen fermentation, rumen microbiome, lipid metabolism, innate immunity, milk production performance, and plasma physiological and biochemical parameters in the post-peripartum period (unpublished). The objective of this study was to assess the effect of NAc supplementation on rumen fermentation parameters and bacterial community composition of Chinese Holstein cows in the post-peripartum period under the same TMR conditions, using high-throughput sequencing techniques.

The fermentation profile of rumen fluid pH, NH_3_-N, MCP and VFA concentrations reflects the function and stability of the internal rumen environment (Zhang et al., 2022). Rumen pH is influenced by the composition of the diet and can reflect rumen fermentation and health (Wang et al., 2020). The increased concentrations of total VFA often lead to a lower rumen pH (Aschenbach et al., 2011; Fregulia et al., 2021). However, in this trial, the increase in total VFA did not change the rumen fluid pH. We speculate that the stability of rumen pH may be related to the weak alkalinity of the aqueous NAc solution, as NAc is a strong base and weak acid salt (Neavyn et al., 2013). Another significant influencing factor is the higher level of NH_3_-N concentration in the rumen, which readily associates with protons to increase pH (Wang and Fung, 1996). The stable rumen fluid pH also suggests that NAc supplementation did not affect the normal function of rumen microorganisms.

In ruminants, nitrogen is shuttled between the portal excretory viscera (particularly the rumen) and the liver in the form of NH_3_-N and urea, a physiological mechanism known as urea-N recycling, which is vital for the utilization of nitrogen in the diet (Lapierre and Lobley, 2001). Ruminal fluid NH_3_-N concentration is influenced by the amount and degradability of dietary and endogenous sources of nitrogen and dietary carbohydrate sources, which reflects changes in the degree of ruminal crude protein degradation (Reynolds and Kristensen, 2008; Li et al., 2019). The use of NH_3_-N for MCP synthesis is energy-dependent (Reynolds, 2006). Therefore, the metabolite of carbohydrates, short-chain fatty acids, can influence the concentration of NH_3_-N. In this experiment, the addition of NAc did not affect NH_3_-N levels but increased the concentration of MCP in the rumen fluid. The MCP is synthesized by microorganisms with NH_3_-N and amino acids using energy to provide endogenous rumen protein, which allows cows to improve crude protein availability (Xie et al., 2019). Furthermore, the results of our additional trial (unpublished) showed that NAc supplementation increased feed intake in cows in the post-peripartum period. Hence, we suggest that NAc supplementation provides more crude protein and carbohydrates to the rumen by promoting feed intake, which increases NH_3_-N production. Microorganisms use the NH_3_-N, peptides and amino acids produced to synthesize MCP, which ultimately leads to increased concentrations of MCP in the rumen. However, whether this process increases the apparent digestibility of crude protein is something we need to verify further.

The VFAs produced by microbial fermentation of carbohydrates and endogenous substrates are an essential source of energy for cows, providing approximately 70% of the total energy required (Bergman, 1990). In this trial, NAc supplementation increased the concentration of rumen TVFA. More TVFA production was able to stimulate an expansion of the rumen absorption area while improving VFA absorption, which was beneficial in alleviating NEB in cows in the post-peripartum period (Melo et al., 2013; Schären et al., 2016; Dieho et al., 2017; Zhang et al., 2022). Moreover, in the NAc supplemented group, the concentrations of acetic, propionic, isobutyric and isovaleric acids were increasing, and there was a trend towards increasing concentrations of butyric and valeric acids. Similarly, a previous study reported that infusion of 36 mol/d acetic acid into cows in the post-peripartum period increased ruminal acetic, isobutyric and isovaleric acid concentrations. The molar ratios of rumen acetic, propionic and butyric acids were all unaltered in this study, unlike the previous report (Sheperd and Combs, 1998). This may be due to the fact that Sheperd et al. collected rumen fluid 2 h after the afternoon feeding when the VFAs concentrations had not reached equilibrium values for fermentation and absorption. In this experiment, the rumen was sampled in the morning before feeding, when rumen VFAs concentrations and molar ratios were in equilibrium. In addition, the addition of NAc did not alter the molar ratio of VFAs in the rumen, which may be related to the increased absorption capacity of the rumen epithelium (Zhang et al., 2022). Acetate is the primary substrate for the *ab initio* synthesis of milk fat in cows and is the main VFA produced by the rumen, which provides 45% of the energy generated by VFA metabolism (Baldwin and Smith, 1971; Bergman, 1990). Recent studies have suggested that acetate supplementation increases milk fat production primarily by increasing mammary gland neonatal lipogenesis (Urrutia and Harvatine, 2017; Urrutia et al., 2019; Matamoros et al., 2021). However, it is unclear whether the use of acetate affects rumen fermentation patterns in dairy cows. Acetic acid is rapidly absorbed in the rumen, and the molar ratio of acetic acid may increase slightly after feeding, but there is no change at the time of sampling. Our results showed that supplementation with NAc increased rumen fluid TVFA concentration and kept the molar ratio constant, suggesting that supplementation with NAc promotes rumen fermentation and increases rumen VFA production.

Differences in microbial community composition were mainly attributed to diets (Ley et al., 2008; Henderson et al., 2015). However, there were few previous reports on the effects of NAc supplementation on rumen bacterial communities in dairy cows in the post-peripartum period. The results showed that NAc supplementation did not alter the α-diversity indices (chao1, observed_otus, shannon, simpson, pielou_e), indicating bacterial diversity and richness in the rumen were not affected. The influence of the relative abundance of rumen bacteria is crucial to the apparent digestibility of nutrients and rumen fermentation characteristics, which may significantly affect ruminants’ productive performance and health status (Ran et al., 2021; Zhou et al., 2022). Feeding NAc did not affect the α-diversity of the rumen bacterial community, which explains the unaltered rumen NH_3_-N and VFA molar percentages in the NAc treated group.

In contrast, the PCoA and NMDS results showed that NAc supplementation altered the structure of the rumen bacterial community. In the current results, NAc supplementation changed the relative abundance of the dominant phylum and most genera with relative abundance ≥0.5%. Although evidence that NAc supplementation affects rumen microbial communities in the post-peripartum period is lacking, it has been shown that short-chain fatty acid salt supplementation reduces bacterial diversity and alters rumen microbiota before and after the weaning of calves (Cao et al., 2020). Dietary variation has an important effect on rumen microbial community composition. Still, the ratio of forage to concentrate and the processing method of the diet are the main factors affecting rumen microbial population structure on the same diet basis (Henderson et al., 2015; Bi et al., 2018; Zhou et al., 2022). More studies have shown that high grain diets can alter the structure of the rumen microbiota (Mao et al., 2015; Seddik et al., 2019; Plaizier et al., 2021). Ruminal hydrogenotrophic acetogens (e.g., *Acetitomaculum*) can use both inorganic (H_2_/CO_2_) and organic substrates (e.g., cellobiose and glucose) in the diet as energy and carbon sources to produce acetate. And the molar percentage of acetate and the molar ratio of acetate to propionate is higher in cattle adapted to fibre-rich diets (Joblin, 1999; Kim et al., 2020; Li Q. S. et al., 2022). Our results suggest that the relative abundance of rumen microbiota composition at the genus level is influenced by NAc supplementation. More often than not, the molar ratio of fermentable acids in the rumen fluid remains relatively balanced after acetic acid, a precursor substance for milk fat synthesis, is rapidly absorbed by the cow’s rumen for milk fat synthesis. This study showed that supplementation of NAc in the diet altered the composition of the rumen bacterial community but not the α-diversity, which may have contributed to the increase in the concentration of VFAs in the rumen. At the same time, the molar ratio of VFAs remained unchanged.

The *Bacteroidota*, *Firmicutes*, *Patescibacteria* and *Proteobacteria* were the main gates in the rumen in this study, similar to previous studies (Seddik et al., 2019; Plaizier et al., 2021). The sequence analysis of 16S rRNA showed that *Bacteroidota* was the dominant bacterium after NAc supplementation and consisted mainly of *Prevotella*. Previous reports have demonstrated that *Prevotella* was the dominant genus in the rumen of dairy cows which was similar to the present study results (Mu et al., 2021; Plaizier et al., 2021). *Prevotella* is known to degrade starch and protein and is usually positively correlated with the levels of carbohydrate, resistant starch and fiber in the diet, and has a number of OTUs strongly correlated with feed efficiency (Fregulia et al., 2021; Tett et al., 2021). Furthermore, NH_3_-N concentrations were significantly positively correlated with the relative abundance of *Prevotella* (Cao et al., 2020). In this study, supplementation with NAc increased the relative abundance of *Prevotella*, which explained the greater NH_3_-N production in the rumen. Previous studies have reported that high-fat diets can increase the relative abundance of the *Bacteroidota* (Wan et al., 2019), thereby improving lipid metabolism by regulating acetate production, of which the genus *Rikenellaceae_RC9_gut_group* also promotes lipid metabolism (Yin et al., 2018; Zhou et al., 2018). In the current study, supplementation with NAc increased the relative abundance of *Rikenellaceae_RC9_gut_group*, suggesting a potential role in rumen lipid metabolism. However, the results also showed that the relative abundance of *RF39* and *Clostridia_UCG-014* was lower in the NAc group than in the control group. The *Clostridia_UCG-014* is a pro-inflammatory bacterium, and its high abundance may lead to the production of more pro-inflammatory metabolites, which are detrimental to the organism’s health (Wang et al., 2021; Li M. et al., 2022). Previous studies have shown that the relative abundance of *RF39* and *Clostridia_UCG-014* is negatively correlated with milk protein production and that these bacteria have a negative role in improving rumen nitrogen metabolism. This may indicate that the addition of NAc improves rumen health, promotes nitrogen metabolism and regulates milk fat production by inhibiting *RF39* and *Clostridia_UCG-014* in *Bacteroidota*. Overall, supplementing the same TMR diet with NAc changed the composition of the microbial community at the phylum and genus level but not the α-diversity, which may improve lipid metabolism, nitrogen metabolism and rumen health.

VFAs produced by microbial fermentation of organic matter in the diet cause pH changes that affect the microbial ecosystem. And the change in pH value affects the growth of rumen microorganisms and the type and quantity of fermentation products (Aschenbach et al., 2011). By comparing Spearman correlations between rumen fermentation indicators and bacterial populations, we determined the changes in rumen microbial genus levels induced by NAc supplementation and its effect on rumen fermentation. The main fermentation products of *Prevotella* are acetic and succinic acids and, to a lesser extent, isobutyric, isovaleric and lactic acids, which are usually closely related to feed efficiency (Roager et al., 2019; Fregulia et al., 2021). Previous studies have suggested that significant increases in *Prevotella* are positively associated with voluntary feed intake, growth performance and high energy use efficiency (Zhang et al., 2019; Wu et al., 2022). It has been shown that the supplementation of acetic acid promotes the growth of *Prevotella* by promoting the synthesis of outer membrane proteins and lipid membranes (Trautmann et al., 2020). This may explain why NAc supplementation increased the relative abundance of *Prevotella* in this study, implying a positive feeding effect of NAc supplementation in the post-peripartum period cows. In addition, *RF39* and *Clostridia_UCG_014* were negatively correlated with rumen fluid VFAs and MCP concentrations, which negatively contribute to rumen nitrogen metabolism and can lead to low feed efficiency and milk protein production (Wang et al., 2021; Li M. et al., 2022). Thus, NAc supplementation may improve NEB status in late-periparturient cows by promoting the growth of *Prevotella*, which is associated with high energy efficiency and inhibiting the relative abundance of *RF39* and *Clostridia_UCG_014*, which are negatively associated with feed efficiency, thereby increasing ruminal VFAs concentrations.

In general, there was no significant change in microbial α-diversity, but microbial community composition at the phylum and genus level was altered; no change in the molar ratio of VFAs, but an increase in the concentration of VFAs; this may be due to NAc supplementation promoting rumen fermentation by altering the structure of the flora, on an identical diet TMR basis. However, it is undeniable that rumen microbial community composition does not fully represent the actual effects of NAc supplementation on the rumen of post-partum cows. Therefore, future studies involving metabolomics and *in vitro* trial validation should be considered, and additional directions are needed to explore the specific effects of acetic acid supplementation.

## Conclusion

This study showed that supplementation of NAc to late periparturient cows on the same basal TMR diet altered microbiota and promoted ruminal fermentation. NAc supplementation had no significant effect on the α-diversity of the rumen bacterial community but upregulate the relative abundance of *Prevotella* and downregulate the relative abundance of *RF39* and *Clostridia_UCG_014*. This study will help guide future research into using NAc in alleviating NEB in post-partum period cows.

## Data availability statement

The datasets presented in this study can be found in online repositories. The names of the repository/repositories and accession number(s) can be found at: https://ngdc.cncb.ac.cn/gsa/, PRJCA010405.

## Ethics statement

The animal study was reviewed and approved by the Institutional Animal Care and Use Committee (SYXK (Su) IACUC 2016-0019), Yangzhou University. Written informed consent was obtained from the owners for the participation of their animals in this study.

## Author contributions

ZC and KZ designed the whole experiment and verified the validity of experiment and checked the results. ZC, ZM, and DT performed the experiment, including chemical analysis, and statistical analysis. GZ, OD, and ML worked on the manuscript. ZM, DT, and ML participated in the experiment design and gave valuable advice. All of the authors have read and approved the final version of this manuscript.

## Funding

This study was supported by the National Natural Science Foundation of China (No. 31972589), and the earmarked fund for CARS36.

## Conflict of interest

The authors declare that the research was conducted in the absence of any commercial or financial relationships that could be construed as a potential conflict of interest.

## Publisher’s note

All claims expressed in this article are solely those of the authors and do not necessarily represent those of their affiliated organizations, or those of the publisher, the editors and the reviewers. Any product that may be evaluated in this article, or claim that may be made by its manufacturer, is not guaranteed or endorsed by the publisher.

## Supplementary material

The Supplementary material for this article can be found online at: https://www.frontiersin.org/articles/10.3389/fmicb.2022.1053503/full#supplementary-material

## Footnotes

^1^ https://magic.novogene.com/
