# Supplementary material for: Effects of supplementation of sodium acetate on rumen fermentation and microbiota in postpartum dairy cows
Source: Front Microbiol. 2022 Nov 21;13:1053503. doi: 10.3389/fmicb.2022.1053503 (PMC9720668; doi:10.3389/fmicb.2022.1053503)
Supplement: Supplementary file 1 [file Data_Sheet_1.PDF]

Supplementary Material

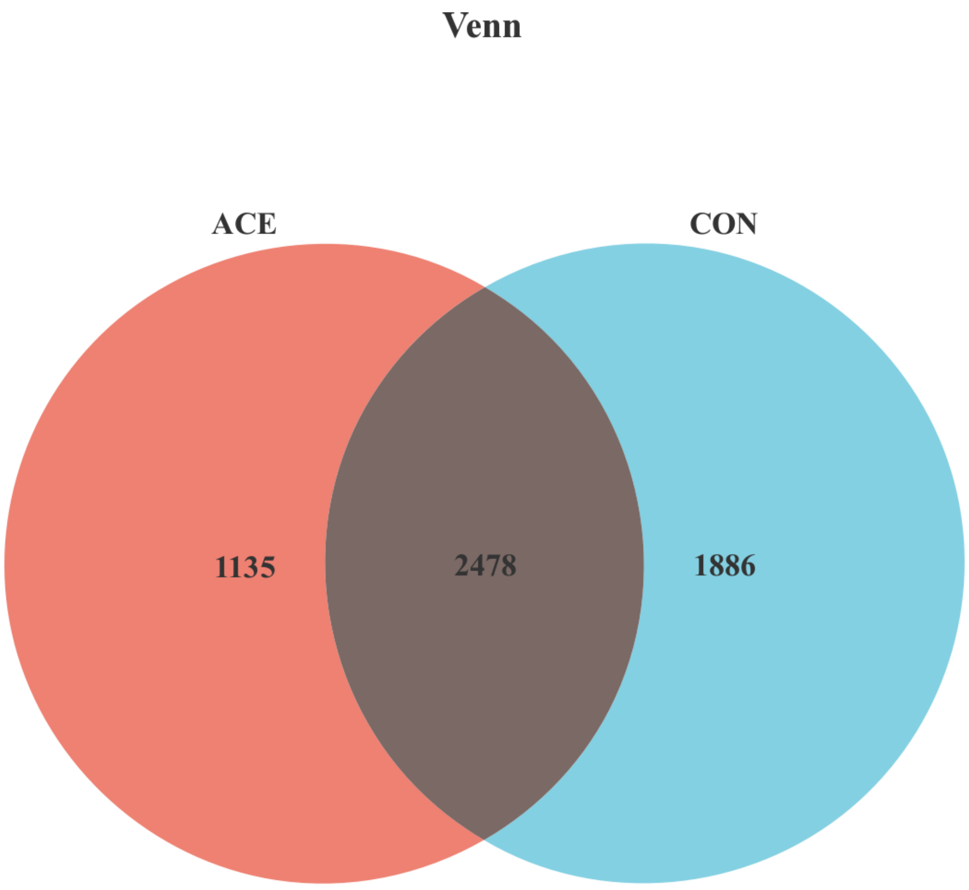

Supplementary Figure S1. Number of common and unique ASVs of rumen bacteria in CON and ACE groups. CON = control group, basal diet plus 468 g/day oral sodium chloride; ACE = NAc group, basal diet plus 656 g/day oral NAc.

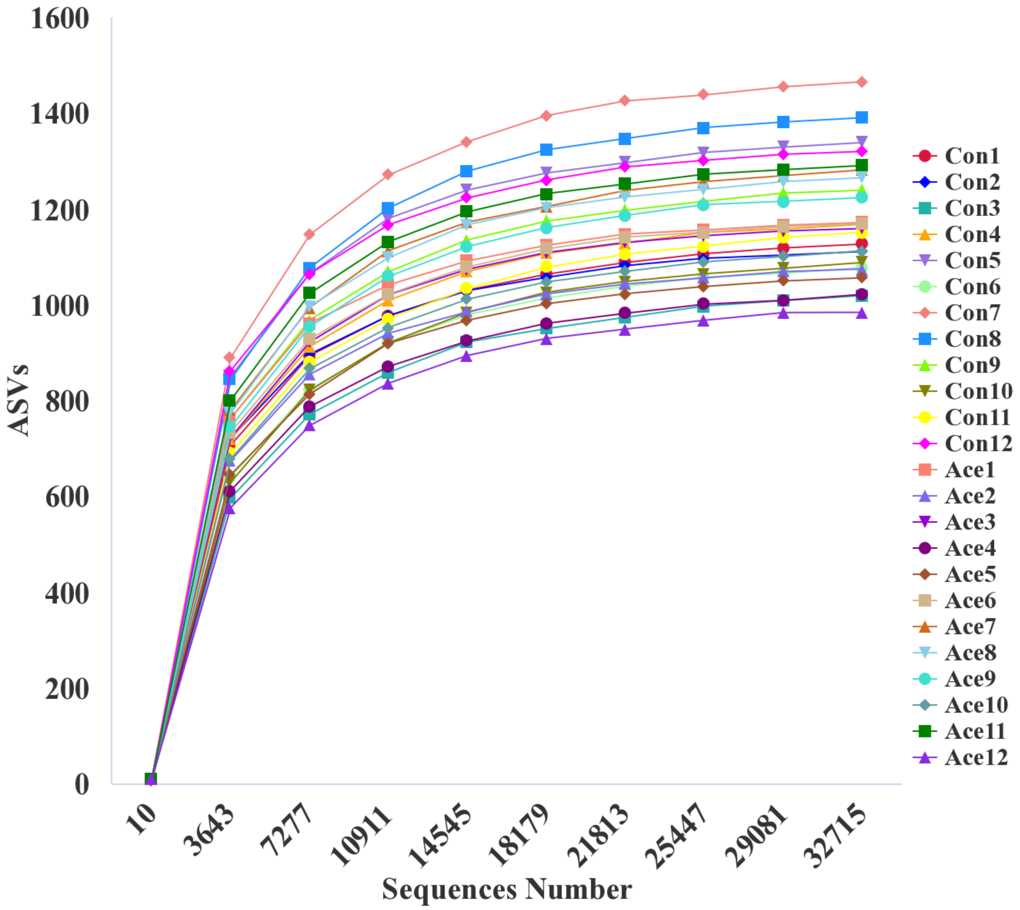

Supplementary Figure S2. Rarefaction curves of ASVs number in rumen fluid samples. CON = control group, basal diet plus 468 g/day oral sodium chloride; ACE = NAc group, basal diet plus 656 g/day oral NAc.

Table S1. Effect of NAc addition on the molar ratio of VFA in rumen fluid of dairy cows in the post-peripartum period.

Items	Treatments[1]		*P*-value
	CON	ACE	
Acetate, (mol/100 mol)	69.95±0.71	70.49±0.42	0.522
Propionate, (mol/100 mol)	17.08±0.58	16.55±0.28	0.424
Iso-butyrate, (mol/100 mol)	0.36±0.01	0.34±0.01	0.264
Butyrate, (mol/100 mol)	10.27±0.56	10.27±0.26	0.997
Iso-valerate, (mol/100 mol)	1.16±0.10	1.23±0.06	0.569
Valerate, (mol/100 mol)	1.18±0.06	1.12±0.08	0.409

[1]Treatments: CON = control group, basal diet plus 468 g/day oral sodium chloride; ACE = NAc group, basal diet plus 656 g/day oral NAc.
